# Thermal Analysis and Operational Characteristics of an AlGaN/GaN High Electron Mobility Transistor with Copper-Filled Structures: A Simulation Study

**DOI:** 10.3390/mi11010053

**Published:** 2019-12-31

**Authors:** Kyu-Won Jang, In-Tae Hwang, Hyun-Jung Kim, Sang-Heung Lee, Jong-Won Lim, Hyun-Seok Kim

**Affiliations:** 1 Division of Electronics and Electrical Engineering, Dongguk University-Seoul, Seoul 04620, Korea; jgw0911@naver.com (K.-W.J.); dltlwhgrh@gmail.com (I.-T.H.); best7hj@daum.net (H.-J.K.); 2 Electronics and Telecommunications Research Institute, Daejeon 34129, Korea; shl@etri.re.kr (S.-H.L.); jwlim@etri.re.kr (J.-W.L.)

**Keywords:** GaN, high electron mobility transistor, self-heating effect, copper-filled structure, thermal conductivity

## Abstract

In this study, we investigated the operational characteristics of AlGaN/GaN high electron mobility transistors (HEMTs) by applying the copper-filled trench and via structures for improved heat dissipation. Therefore, we used a basic T-gate HEMT device to construct the thermal structures. To identify the heat flow across the device structure, a thermal conductivity model and the heat transfer properties corresponding to the GaN, SiC, and Cu materials were applied. Initially, we simulated the direct current (DC) characteristics of a basic GaN on SiC HEMT to confirm the self-heating effect on AlGaN/GaN HEMT. Then, to verify the heat sink effect of the copper-filled thermal structures, we compared the DC characteristics such as the threshold voltage, transconductance, saturation current, and breakdown voltage. Finally, we estimated and compared the lattice temperature of a two-dimensional electron gas channel, the vertical lattice temperature near the drain-side gate head edge, and the transient thermal analysis for the copper-filled thermal trench and via structures. Through this study, we could optimize the operational characteristics of the device by applying an effective heat dissipation structure to the AlGaN/GaN HEMT.

## 1. Introduction

AlGaN/GaN high electron mobility transistors (HEMTs) are used as power-amplifying devices because of their advantages, such as high breakdown voltage, wide bandgap, and stability at high temperatures of up to approximately 1000 K [1,2,3,4]. GaN based devices are still operational even if they exhibit deterioration and unstable behavior at high temperatures. However, the self-heating effect (SHE), which causes a gate leakage current, breakdown voltage degradation, and a negatively sloped saturation curve, has become a major issue in power amplification devices [5,6,7,8,9]. To be specific, phonon scattering enhanced by SHE degrades the direct current (DC) and radio frequency (RF) characteristics of HEMTs. Therefore, to control such influences, research on field plates, high thermal conductivity materials, and air–water cooling systems has been actively conducted [10,11,12,13,14].

To propose an optimized thermal structure for the heat sink, we simulated the DC and thermal characteristics of AlGaN/GaN HEMTs by considering the application of copper-filled structures to a SiC substrate. Two different thermal structures, namely a copper-filled thermal trench (CTT) and a copper-filled thermal via (CTV), were used [15,16,17,18,19,20,21]. To verify the exact heat sink effect, we applied the thermal conductivity model and the corresponding parameters for each material. To investigate the electrical operational degradation caused by SHE, we first simulated the thermal effects on the DC characteristics of a conventional basic GaN on SiC (BGS) HEMT structure. The DC characteristics of the BGS structure were then compared with those of the CTT and CTV structures. Additionally, steady-state thermal characteristics, such as the lateral lattice temperature inside the two-dimensional electron gas (2-DEG) channel and the vertical lattice temperature during device operation followed by a transient thermal analysis were discussed.

## 2. Materials and Methods

**Figure 1:**
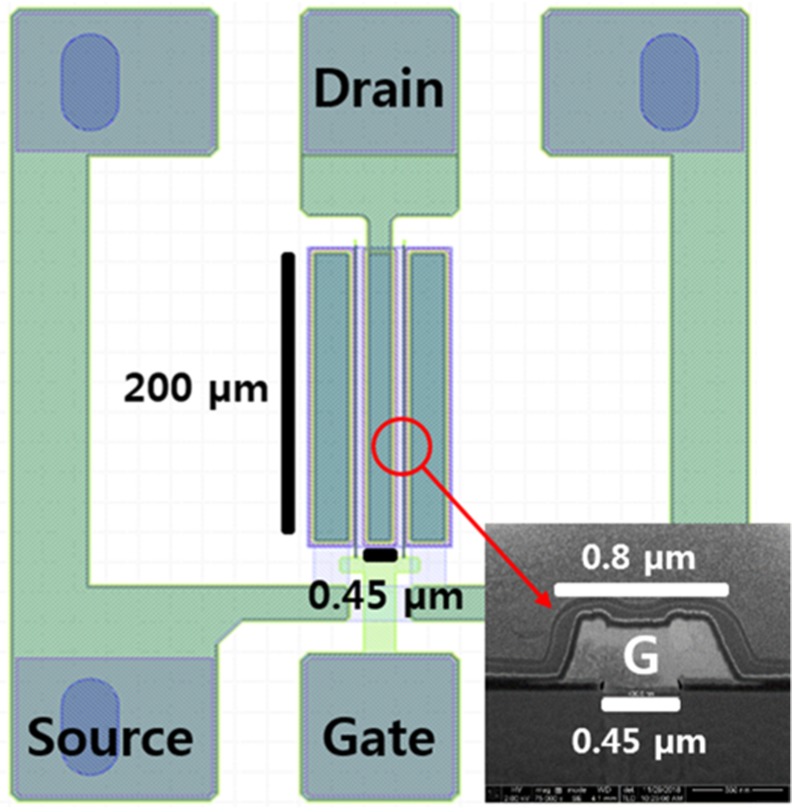
Top-view schematic of the two-finger basic AlGaN/GaN high electron mobility transistor (HEMT) structure and the cross-sectional scanning electron microscope (SEM) image of the one-finger gate electrode.

Figure 1 shows a top schematic view of the two-finger BGS structure for the fabrication consisting of the drain, gate, and source contact pads. The gate and source electrodes were patterned symmetrically on both sides of the drain electrode in the center. The inset shows a cross-sectional scanning electron microscope (SEM) image of the T-gate with a gate length of 0.45 μm, a head length of 0.8 μm, and a width of 200 μm.

**Figure 2:**
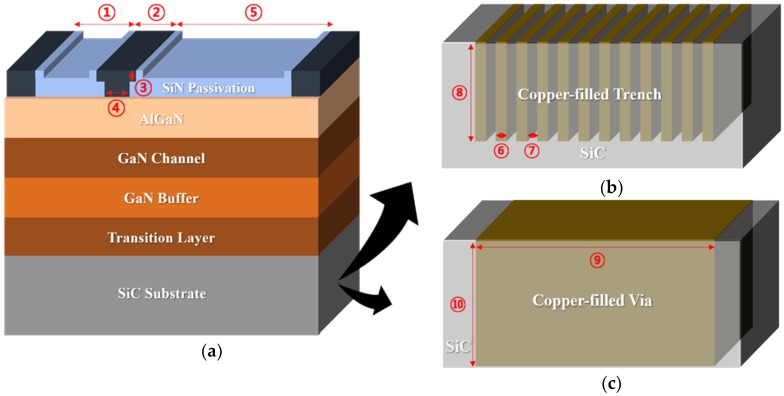
AlGaN/GaN HEMT structure: (**a**) basic AlGaN/GaN on SiC, (**b**) copper-filled thermal trench, and (**c**) copper-filled thermal via in the SiC substrate.

To investigate the thermal effect on the AlGaN/GaN HEMT, two different copper-filled thermal structures were applied under the active region of the BGS HEMT, as shown in Figure 2. Figure 2a shows a unit BGS device. Furthermore, the two thermal structures in the SiC substrate are depicted in Figure 2b,c. The SiC substrate under the active region was etched away and filled with copper, forming trenches or via. Table 1 provides detailed geometrical information of the BGS, CTT, and CTV structures. To fabricate vias and trenches in the SiC substrate, the base metal Ti and Au were first deposited across the SiC wafer as adhesion layers for nickel etch mask deposition. The deposition thickness of Ti and Au were 3 nm and 8 nm, respectively. Afterwards, to open up the SiC substrate surface, an etching window was selectively defined with photolithography, followed by a lift-off process after nick etch mask deposition. Before SiC etching, removal of the base metal was performed by Ar-based inductively-coupled plasma reactive-ion-etching (ICP RIE). Then, SiC etching was performed by SF_6_-based ICP RIE using a high RF power of above 2000 W. The etch rate of SiC was 1.6 µm/min. The nickel etch mask was removed in a diluted HNO_3_. Then, the base metals, Au and Ti, were sequentially removed using iodine-based etchant and buffered oxide etchant (BOE), respectively. Finally, the device was immersed in a diluted HCl solution and ultrasonically cleaned simultaneously to remove etch residues resulting from the etching process. Next, the trench and via structures were filled with copper by an electroplating and a damascene process. In the case of the via structure, copper-filled via formation could be performed from the backside. However, there was an issue in the deposition of the GaN layer for CTT. Since the transition layer and the GaN layer must be deposited after the formation of the trench structure from the top SiC substrate, the epitaxial growth of the GaN on copper using metal organic chemical vapor deposition (MOCVD) had interfacial reaction problems between the GaN and the copper because of high temperature. However, this interfacial problem between the GaN and copper could be resolved by using a low temperature growth of the pulsed laser deposition (PLD) method [22,23,24]. Moreover, growth of III nitrides using PLD was possible at even room temperature due to the enhanced kinetic energies of the film precursors, which promoted surface transferal.

Acceptor trap doping was applied in the GaN buffer layer to prevent the substrate leakage current. We used the Gaussian acceptor doping profile, in which the doping concentration gradually decreased so that the acceptor doping concentration in the interface of the AlGaN and the GaN layer was set to less than $10^{15}/{\mathrm{cm}}^{3}$. More specifically, the concentration at this interface was $6.82\times 10^{14}/{\mathrm{cm}}^{3}$ [25]. In this simulation study, the same physical values for electron sheet density and electron mobility were assigned for all the device epitaxy layers. The other material parameters of GaN and AlGaN used for the simulation are summarized in Table 2.

In order to consider the overall thermal effect for the DC operational characteristics of AlGaN/GaN HEMT, SHE and the thermal conductivity model were required for the calculation. The SHE model included the lattice heat flow and heat generation equations [26,27]. Thus, we defined the lattice heat flow as follows:

(1) $$C\frac{\partial T_{L}}{\partial t}=\nabla \left(k\nabla T_{L}\right)+H,$$

where *C* is the heat capacitance per unit volume, *T_L_* is the local lattice temperature, *k* is the thermal conductivity, and *H* is the heat generation, which can be defined as follows:

(2) $$H=\left(\frac{{\left|\vec{J_{n}}\right|}^{2}}{q\mu_{n}n}+\frac{{\left|\vec{J_{p}}\right|}^{2}}{q\mu_{p}p}\right)+q\left(R-G\right)\left[\varphi_{p}-\varphi_{n}+T_{L}\left(P_{p}-P_{n}\right)\right],$$

where $\left(\frac{{\left|\vec{J_{n}}\right|}^{2}}{q\mu_{n}n}+\frac{{\left|\vec{J_{p}}\right|}^{2}}{q\mu_{p}p}\right)$ is the Joule heating term, and $q\left(R-G\right)\left[\varphi_{p}-\varphi_{n}+T_{L}\left(P_{p}-P_{n}\right)\right]$ is the recombination and generation heating and cooling term.

**Figure 3:**
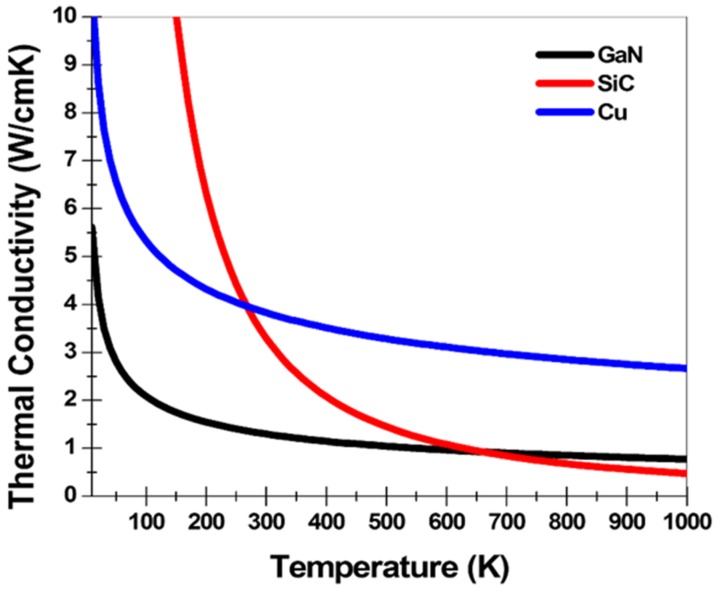
Thermal conductivity profiles for the GaN, SiC, and Cu materials.

Figure 3 represents the thermal conductivity as a function of the lattice temperature corresponding to each GaN, SiC, and Cu. The thermal conductivity model applied to the simulation can be expressed as follows:

(3) $$k\left(T_{L}\right)=\left(TC.CONST\right)/{\left(T_{L}/300\right)}^{TC.NPOW}$$

where *TC.CONST* is a thermal conductivity constant of each material for 300 K and *TC.NPOW* is an experimental value of each material for the thermal conductivity model. Table 3 shows the thermal constants used for the thermal conductivity model for each material. As shown in Figure 3, at temperatures from 300 K to approximately 650 K, the thermal conductivity was high in the order of Cu, SiC, and GaN [28,29,30].

**Figure 4:**
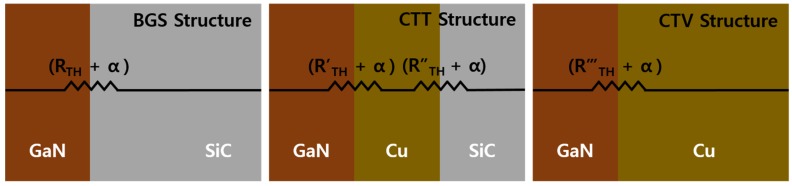
Thermal boundary resistances for basic GaN on SiC (BGS), copper-filled thermal trench (CTT), and copper-filled thermal via (CTV) structures.

Thermal boundary resistances (TBRs) are significant factors for heat transfer characteristics analysis. Since the process methods and conditions for each structure are different, TBRs for each structure will be also different. Therefore, in the simulations, the dynamic TBRs (R_TH_, R’_TH_, R’’_TH_, and R’’’_TH_) were calculated by the differences of the thermal conductivity corresponding to the lattice temperature for each material as depicted in Figure 4. The dynamic TBRs for three structures were considered by the heat flow equation and the thermal conductivity model. In addition, for the effective heat flow analysis, we applied the static TBR (α) of $2.5\times 10^{-8}\;m^{2}K/W$, which is within the actual range measured at the interface between any material and GaN [31,32]. Moreover, in order to carry out the simulation considering the convection heat transfer, we designated a thermal boundary condition with an external temperature of 300 K at the bottom of all structures. In addition, for the basic physical calculations, the Shockley–Read–Hall (SRH) recombination, the Auger recombination, and the Fermi–Dirac distribution function were applied to the simulation. Furthermore, the Selberherr impact ionization model, which is the temperature dependent impact ionization model, was used to calculate the impact ionization process near the gate edge of the device [33,34].

## 3. Results and Discussions

First of all, in order to have confidence in simulation reliability, we tried to match simulation values with experimental data for I-V transfer characteristics by applying material parameters and physical models, including the self-heating effect.

**Figure 5:**
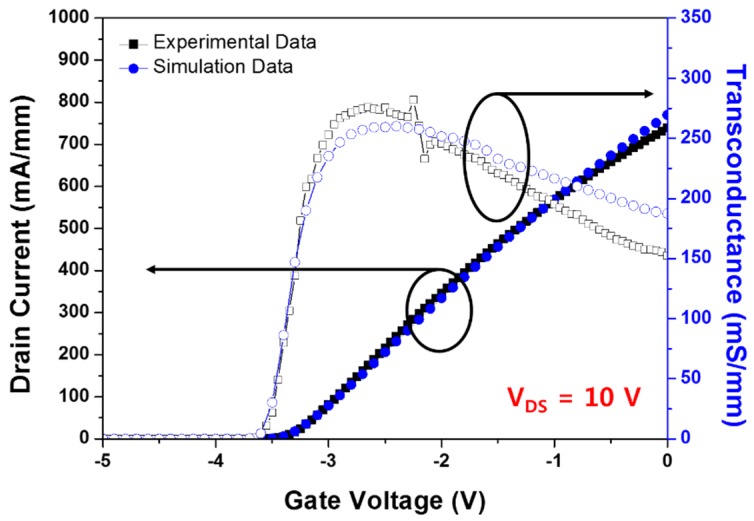
Simulation and experimental data for I-V transfer characteristics considering the self-heating effect (SHE) at V_DS_ = 10 V.

The matching process of the I-V transfer characteristics for drain voltage = 10 V was preceded. In the case of experimental data, the BGS HEMT’s drain current and transconductance were measured using Yokogawa GS200 and Keithley 2410 DC bias measurement systems in a probe station at room temperature. Thereafter, we calculated the electrical and thermal properties of GaN HEMT based on the simulation and investigated their tendency. Figure 5 shows the simulation and experimental values for I-V transfer characteristics at drain voltage = 10 V. We extracted the simulation conditions from the experimental data.

**Figure 6:**
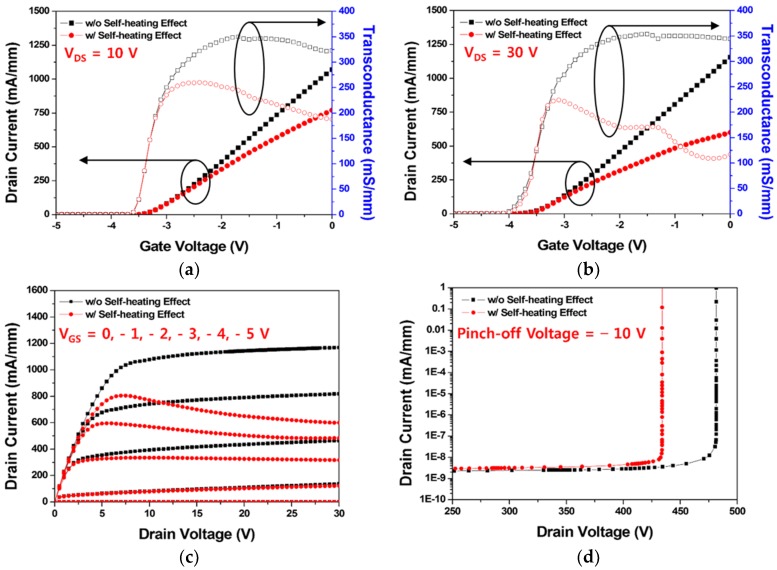
Direct current (DC) characteristics of the BGS HEMT structure with and without the SHE: I–V transfer characteristics at (**a**) V_DS_ = 10 V and (**b**) V_DS_ = 30 V; (**c**) saturation current when gate voltages were −5, −4, −3, −2, −1, and 0 V; and (**d**) breakdown characteristics at a pinch-off of V_GS_ = −10 V.

It was necessary to confirm the degradation due to the SHE in the operational characteristics of the AlGaN/GaN HEMTs. As shown in Figure 6a,b, the I–V transfer characteristics, such as threshold voltage, drain current, and transconductance for the drain voltage of 10 V and 30 V, were compared with and without the SHE. Consequently, the SHE decreased both the drain current and the transconductance as a whole, and this tendency became more apparent as the drain voltage increased. Accordingly, we verified that the higher electric field between the drain and the source resulted in the lesser drain current because of the higher SHE. However, the threshold voltage did not change. Figure 6c shows the saturation current characteristics for the gate voltages of −5, −4, −3, −2, −1, and 0 V. With the application of the SHE, the overall drain current decreased, and the saturation curve showed a negative slope. The heat generated by the applied electric field and current density caused a thermal scattering. This effect reduced electron mobility. Therefore, as the applied drain voltage increased, the carrier scattering effect increased, reducing the drain current density. For this reason, the drain current in the saturation region showed a negative slope as the drain voltage increased. The breakdown voltage with a gate pinch-off voltage of −10 V, as shown in Figure 6d, also decreased from 481.7 V to 434.2 V because of the SHE. From the overall electrical perspective, the SHE severely degraded the DC operational characteristics of the GaN HEMT.

**Figure 7:**
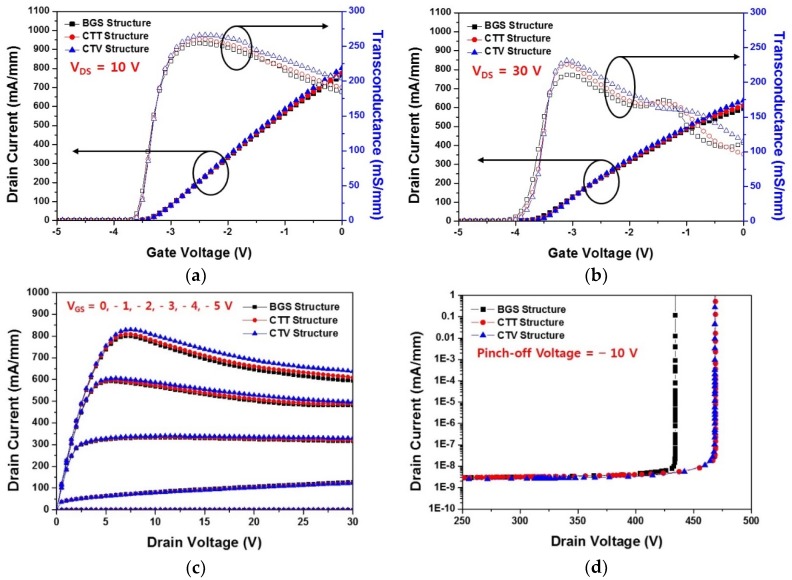
Comparison of the DC characteristics considering the SHE for the BGS, CTT, and CTV structures: I–V transfer characteristics at (**a**) V_DS_ = 10 V and (**b**) V_DS_ = 30 V; (**c**) saturation current when gate voltages were −5, −4, −3, −2, −1, and 0 V; and (**d**) breakdown characteristics at a pinch-off of V_GS_ = −10 V.

To control the heat generated by the SHE, we used two different copper-filled thermal structures to the SiC substrate of the BGS HEMT. We simulated the DC characteristics of the two copper-filled thermal structures with the SHE and compared them to those of the BGS HEMT. Figure 7 shows the comparison of the DC characteristics with the SHE for the BGS, CTT, and CTV structures. Figure 7a,b shows the I–V transfer characteristics for all the three structures simulated when the drain voltage was 10 V and 30 V, respectively. The overall drain current and the maximum transconductance improved because of the application of the copper-filled thermal structures. CTV showed better improvement than CTT, irrespective of the drain voltage. As shown in Figure 7c, the saturation current characteristics of all the structures were estimated when the gate voltages were −5, −4, −3, −2, −1, and 0 V. The application of thermal structures increased the overall drain current and stabilized the saturation current characteristics as compared to the BGS structure. Figure 7d shows the breakdown voltage characteristics for three structures. The CTT and CTV structures had an enhanced breakdown voltage as compared to the BGS structure. However, the breakdown voltage behavior was similar for the CTT and CTV structures. The breakdown voltages extracted at the point where the drain current was 1 mA/mm were 434, 469, and 468 V for the BGS, CTT, and CTV structures, respectively. One of several operational instabilities due to the SHE was the increase in the gate leakage current, which caused a breakdown of the device. As the operating temperature of the device increased, the gate leakage current increased, which in turn reduced the breakdown voltage. However, the additional heat sinks obtained by the copper filling approach improved breakdown voltage by reducing the gate leakage current as a result of lowering the overall lattice temperature inside the device’s 2-DEG channel. As a result, the application of the copper-filled thermal structures improved the overall DC characteristics. Of these, the CTV structure showed the most thermally optimized GaN HEMT electrical characteristics.

**Figure 8:**
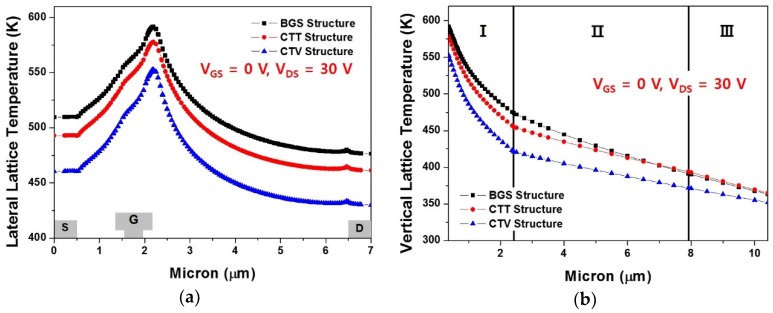
Comparison of the lattice temperature for the BGS, CTT, and CTV structures when V_GS_ = 0 V and V_DS_ = 30 V: (**a**) lateral lattice temperature in the two-dimensional electron gas (2-DEG) channel and (**b**) vertical lattice temperature near the drain-side gate head edge at the point with the highest lateral lattice temperature.

Figure 8 shows the lateral and vertical lattice temperatures for the BGS, CTT, and CTV structures. The highest lateral lattice temperature inside the 2-DEG channel when V_GS_ = 0 V and V_DS_ = 30 V appeared near the drain-side gate head edge for all the three structures, as shown in Figure 8a. The application of copper-filled thermal structures as an additional heat sink had a positive effect in reducing the overall heat generated during device operation. The overall lateral lattice temperature was lower in the order of the CTV, CTT, and the BGS structure, and the peak temperatures were 552.8 K, 577.8 K, and 592.7 K, respectively. Figure 8b shows the vertical lattice temperature near the drain-side gate head edge in the device at the point with the highest lateral lattice temperature from the 2-DEG channel to the bottom of the device. For all three structures, region I was the GaN layer under the 2-DEG channel. Regions II and III were the SiC substrate for the BGS structure. In the CTT structure, region II was a copper-filled thermal trench area, and region III was the SiC substrate, as depicted in Figure 2b. In the CTV structure, regions II and III were both copper-filled thermal via areas. As shown in Figure 8b, the temperature drop across region I was larger in the order of the CTV, CTT, and the BGS structure. Moreover, the overall temperature reduction was more effective for CTV.

**Figure 9:**
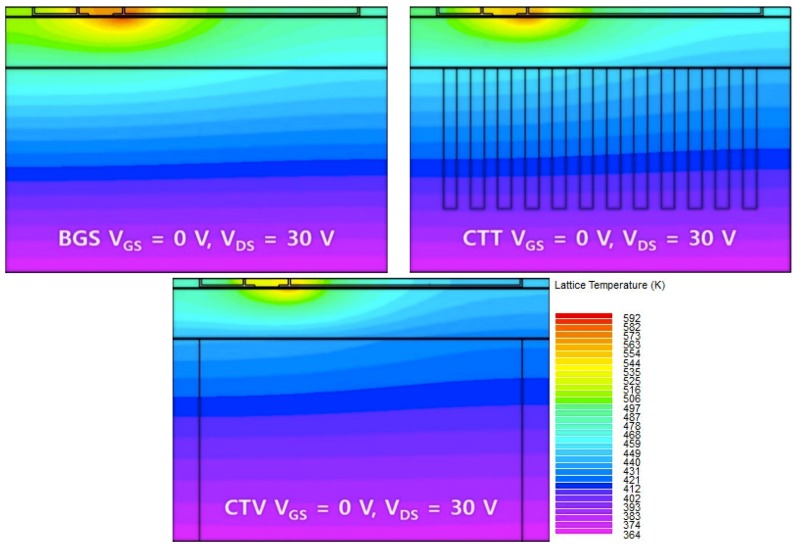
Temperature gradient profiles for the BGS, CTT, and CTV structures when V_GS_ = 0 V and V_DS_ = 30 V.

Figure 9 represents the hot points of three different HEMT structures for gate voltage = 0 V and drain voltage = 30 V using the thermal analysis technology computer-aided design (TCAD) simulator. The CTV structure showed the lowest temperature near the gate electrode. The thermal distribution throughout the GaN HEMT was alleviated the most in CTV.

**Figure 10:**
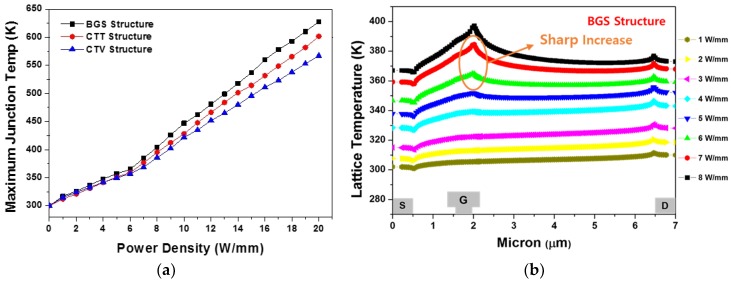
Steady-state thermal properties as a function of the drain power density: (**a**) maximum junction temperature for the BGS, CTT, and CTV structures and (**b**) lattice temperature of the BGS structure inside the 2-DEG channel.

Figure 10a shows the maximum junction temperature across the GaN HEMT for the BGS, CTT, and CTV structures, while the power density increased from 0 to 20 W/mm with a step of 1 W/mm. The increment of temperature for 1 W/mm was approximately 16.8 K, 15.1 K, and 13.6 K on average for the BGS, CTT, and the CTV structure, respectively. This confirmed that the heat generated by the increase in the power density was well controlled in the CTV structure. As shown in Figure 10a, the maximum junction temperature slope changed after a power density of 6 W/mm. The lattice temperature inside the 2-DEG channel for increasing the power density from 1 W/mm to 8 W/mm at 1 W/mm interval is shown in Figure 10b. The temperature peak was extracted at the drain electrode edge at the power density of less than 6 W/mm. However, the temperature peak was extracted near the drain-side gate head edge at the power density higher than 6 W/mm. When the power density increased from 6 W/mm to 7 W/mm, the heat generation near the drain-side gate head edge sharply increased. For this reason, the maximum junction temperature slope suddenly changed after 6 W/mm power density.

**Figure 11:**
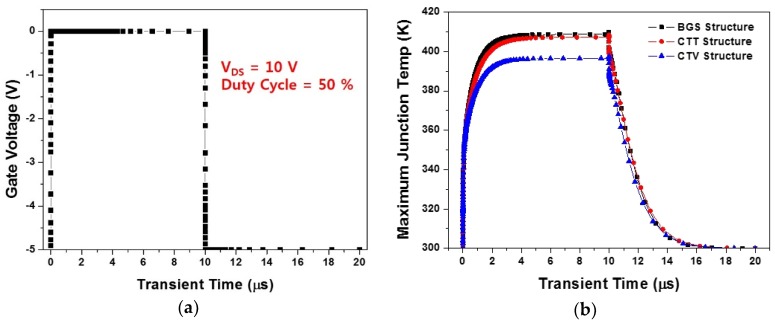
Transient thermal responses in the BGS, CTT, and CTV structures under 50% duty cycle: (**a**) Transient bias condition with a 50% duty cycle and (**b**) transient maximum junction temperature as a function of time.

Furthermore, instead of an analysis of the steady-state thermal characteristics, we conducted the transient thermal analysis of the AlGaN/GaN HEMT to identify the change in the thermal response after the device was turned on and off [35,36,37]. As shown in Figure 11a, for drain voltage = 10 V, a transient bias condition from −5 V to 0 V was applied as the gate voltage. As all the structures took approximately 6–7 μs to reach the thermal equilibrium after turning on, we set both the on- and off-interval to 10 μs. The transient thermal characteristics were simulated with an input bias condition of a 50% duty cycle. Figure 11b shows the transient thermal responses of the BGS, CTT, and CTV structures. As a result, the heating time required to reach the maximum temperature after turning the device on was 4.38 μs for the BGS structure, 4.64 μs for the CTT structure, and 4.42 μs for the CTV structure. The cooling time was almost the same for three structures.

The CTV HEMT, in contrast to the CTT HEMT, was the most thermally optimized HEMT from electrical and thermal points of view. As shown in Figure 3, thermal conductivity between SiC and copper was significantly different at temperatures above 300 K. According to the basic principle of heat transfer, the higher the thermal conductivity of material, the more effective the heat transfer will be. Thus, the CTV structure showed more effective heat transfer than the CTT structure with a large Cu/SiC surface-to-volume ratio.

## 4. Conclusions

In general, the field plate technique can be used to redistribute the heat and electric field concentrated near the drain-side gate head edge, thereby improving the breakdown DC characteristics of AlGaN/GaN HEMTs. However, in this study, the overall lattice temperature inside the 2-DEG channel was reduced by using copper-filled thermal structures. The drain current, maximum transconductance, breakdown voltage, and saturation current characteristics improved when we constructed the two thermal structures. In addition, the application of the thermal structures was an effective method of controlling steady-state thermal characteristics, such as the lateral lattice temperature inside the 2-DEG channel, the vertical lattice temperature, and the heat generation rate with increasing power density. Through a transient thermal analysis, we confirmed that the maximum junction temperatures were lower for the copper-filled thermal structures, and the time to reach the maximum lattice temperature was further slowed down by applying thermal structures to GaN HEMTs. The simulation results suggest that the CTV could improve the thermal management of GaN HEMTs.

## Figures and Tables

**Table 1:** Geometrical parameters for basic GaN on SiC (BGS), copper-filled thermal trench (CTT), and copper-filled thermal via (CTV) structures.

Parameter	Unit	Value
① L_Gate-Source_	μm	1.025
② L_Gate-Head_	μm	0.8
③ L_Gate-Foot_	nm	250
④ L_Gate_	μm	0.45
⑤ L_Gate-Drain_	μm	4.525
⑥ L_CTT-Cu-width_	μm	0.25
⑦ L_Cu-Cu_	μm	0.25
⑧ L_CTT-Cu-depth_	μm	5.5
⑨ L_CTV-Cu-width_	μm	6
⑩ L_CTV-Cu-depth_	μm	8
SiN passivation (AlGaN interface)	nm	90
SiN passivation (gate and side wall)	nm	40
AlGaN	nm	25
GaN channel	nm	50
GaN buffer	μm	2
Transition layer	nm	50
SiC substrate	μm	8

**Table 2:** Material properties used for calculation.

Parameter	Unit	GaN	AlGaN
Bandgap energy	eV	3.39	3.87
Electron affinity	eV	4	2.73
Relative permittivity	-	9.5	9.38
Low field electron mobility	cm^2^/V·s	1460	300
High field mobility model	-	GANSAT Mobility Model	
Electron saturation velocity	cm/s	1.9 × 10^7^	1.12 × 10^7^
Hole saturation velocity	cm/s	1.9 × 10^7^	1.0 × 10^6^
Shockley–Read–Hall (SRH) life time	s	1.0 × 10^−8^	1.0 × 10^−8^

**Table 3:** Thermal constants used for the thermal conductivity model.

Parameter	Unit	GaN	AlGaN	SiC	Cu
Thermal conductivity constant (*TC.CONST*)	W/cm·K	1.3	0.4	3.3	3.83
Thermal conductivity factor (*TC.NPOW*)	-	0.43	0	1.61	0.3
